# Recruitment of Mesenchymal Stem Cells to Damaged Sites by Plant-Derived Components

**DOI:** 10.3389/fcell.2020.00437

**Published:** 2020-06-09

**Authors:** Akito Maeda

**Affiliations:** Skin Regeneration, PIAS Collaborative Research, Graduate School of Pharmaceutical Science, Osaka University, Suita, Japan

**Keywords:** mesenchymal stem cells, cell migration, plant-derived components, cell therapy, tissue repair

## Abstract

Mesenchymal stem cells (MSCs) are capable of differentiating into a limited number of diverse cells and secrete regenerative factors that contribute to the repair of damaged tissue. In response to signals emitted by tissue damage, MSCs migrate from the bone marrow and area surrounding blood vessels within tissues into the circulating blood, and accumulate at the site of damage. Hence, MSC transplantation therapy is beginning to be applied to the treatment of various intractable human diseases. Recent medicinal plants studies have shown that plant-derived components can activate cell functions. For example, several plant-derived components activate cell signaling pathways, such as phosphatidylinositol 3-kinase and mitogen-activated protein kinase (MAPK), enhance expression of the CXCL12/CXCR4 axis, stimulate extracellular matrix remodeling, and consequently, promote cell migration of MSCs. Moreover, plant-derived components have been shown to promote recruitment of MSCs to damaged tissues and enhance healing in disease models, potentially advancing their therapeutic use. This article provides a comprehensive review of several plant-derived components that activate MSC migration and homing to damaged sites to promote tissue repair.

## Introduction

Mesenchymal stem cells (MSCs) are capable of differentiating into a limited yet diverse range of cells, and secrete regenerative factors that contribute to the repair of damaged tissues (Uccelli et al., 2008). MSCs in humans and animals have numerous characteristics, including the expression of specific cell surface markers, some of which are used as criteria for the detection of MSCs in tissues. The International Society for Cellular Therapy proposed that human MSCs should be identified according to positive expression for CD73, CD90, and CD105, and negative for CD11b or CD14, CD19 or CD79α, CD34, CD45, and HLA-DR expression (Dominici et al., 2006). MSCs have been identified in several tissues, including the bone marrow, adipose tissue, cord blood, placenta, and pulp (Pittenger et al., 2019). MSCs have also been reported to migrate to wound sites during the healing process (Wu et al., 2010). Specifically, MSCs have been shown to move from the perivascular area of tissue into the blood circulation in response to signals emitted after tissue damage, and subsequently MSCs in the blood circulation may accumulate in damaged tissue (Rochefort et al., 2006; Iinuma et al., 2015). Thus, MSCs exhibit homing properties, which allows for their spontaneous accumulation at the site of injury.

It has been shown that methods capable of promoting the migration and homing of MSCs to damaged tissue enhance their effect in cell therapy (Park et al., 2015). These models have been reported to implement several approaches including the preconditioning of MSCs, recombinant MSCs, engineering of cell surface proteins of MSCs, and modification of target tissues (Becker and Riet, 2016). To assess MSC migration *in vitro*, either the Boyden chamber method or the Transwell method have been used, which examine movement between concentration gradients, created by separating active substances with a permeable membrane, or by the Scratch wound method using movement in two dimensions, respectively (Justus et al., 2014). Alternatively, for the detection of MSC migration *in vivo*, researchers have developed MSCs labeled with fluorescent dyes, magnetic substances, and radioactive substances, or genetically modified MSCs that express reporters, which are transplanted into a living organism (Krueger et al., 2018). Recently, several clinical trials using cell therapy to transfer exogenous MSCs into the body for treatment of various types of tissue damage and diseases, have demonstrated many advantages and are nearing completion [clinical trials databases: www.clinicaltrials.gov, 306 studies using MSCs with study completed status in February 2020]. The application of MSC therapy has been reported for a broad range of disorders, including various skin diseases, bone defects, cardiac disorders, and brain damage (Kim et al., 2017; Wang et al., 2018; Ward et al., 2018; Presen et al., 2019). In addition, advanced research has been conducted to develop MSCs as cell vectors for application in cancer treatment (Mohr and Zwacka, 2018). Therefore, new materials that enhance the recruitment of transplanted MSCs into damaged tissues, may further improve their therapeutic effect.

Cell migration is stimulated by chemokines, cytokines, growth factors, and other biomolecules, through a number of molecularly and pharmacologically defined signaling pathways (Marquez-Curtis and Janowska-Wieczorek, 2013). MSC migration has been shown to be activated by factors such as CXCL12 (SDF-1), MIP-1, HGF, VEGF, and PDGF. Moreover, specific signaling pathway molecules including CXCR4, phosphatidylinositol 3-kinase (PI3K), mitogen-activated protein kinase (MAPK), and small G proteins have been reported to be involved in the regulation of MSC migration. It has also been reported that hypoxic preconditioning stimulates the expression of genes involved in cell migration, thereby activating MSC migration (Rochefort et al., 2006; Meng et al., 2018). Upon reaching the target site, MSCs can degrade the intercellular matrix and use as a scaffold to migrate through tissue.

Medicinal plants have historically been used in folk medicine to relieve various symptoms and diseases. Studies of compounds derived from medicinal plants have shown that they can molecularly activate various functions in cells and tissues (Shedoeva et al., 2019). In fact, it has been reported that plant-derived components can activate signal transduction pathways related to cell motility (Cho et al., 2014). Although there are few reports concerning plant-related components involved in MSC migration, they represent a promising class of potential candidates to enhance the therapeutic effects of MSC therapy. Nevertheless, it has been reported that plant-derived components activate MSC migration through specific signaling pathways, mobilize MSCs to injured organs, and have healing effects. Moreover, such information may be useful for identifying more effective chemical compounds in the future. Therefore, this article reviews plant-derived components that have been shown to activate MSC migration, and homing, to damaged sites in animal models, where they contribute to the healing process.

## Activation of MSC Migration by Plant-Derived Components

**FIGURE 1:**
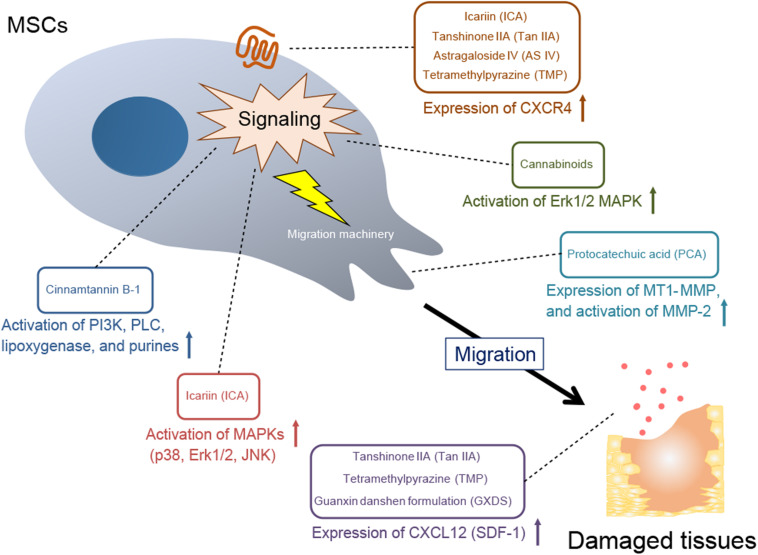
A mechanistic overview of MSC recruitment to damaged sites by plant-derived components. Expression of CXCR4, a receptor for the chemokine CXCL12; activation of specific signaling molecules; expression and activation of MMPs, which are a part of the MSC migration machinery; and expression of CXCL12 (SDF-1) in damaged target tissues are stimulated by plant-derived components, leading to MSC migration.

Several studies have reported that plant-derived components promote MSC migration, which may contribute to healing of damaged tissue (Table 1). This effect is triggered by stimulating mechanisms involved in MSC migration (Figure 1 and Table 2). Below we discuss the implicated plant-derived components in detail.

**TABLE 1:** Plant-derived components promote MSC migration and healing in animal models.

**Component**	**MSC source**	**Assessment of MSC migration**	**Therapeutic model and Administration method**	**References**
Protocatechuic acid (PCA)	AMSC	Transwell method	–	Wang et al., 2008
Cinnamtannin B-1	BMSC	Boyden chamber method, FACS analysis for MSC marker, Detection of luc-expressing MSC	Transdermal administration to the mouse model of skin wound healing	Fujita et al., 2015
Cannabinoids	AMSC, BMSC	Boyden chamber method, Immunofluorescent analysis of MSC	Implantation of the component-loaded microspheres in the rat model of bone-defect	Schmuhl et al., 2014; Kamali et al., 2019
Icariin (ICA)	BMSC	Transwell method, Scratch wound method, Detection of BrdU-labeled MSC	Preconditioning MSCs were injected intravenously into the rabbit model of cartilage-defect	Jiao et al., 2018; Zhu et al., 2018
Tanshinone IIA (Tan IIA)	BMSC	Transwell method, Detection of Dil-labeled MSC	Oral administration to the rat model of acute myocardial infarction	Tong et al., 2011
Astragaloside IV (AS IV)	BMSC	Transwell method, Detection of Dio-labeled MSC	Preconditioning MSCs were injected intravenously into the rat model of acute myocardial infarction	Xie et al., 2013
Tetramethylpyrazine (TMP)	BMSC	Transwell method, Detection of BrdU-labeled MSC	Preconditioning MSCs were injected intravenously into the rat model of cerebral ischemia, or Intraperitoneal administration to the model	Li et al., 2017a, 2019
Guanxin danshen formulation (GXDS) (Including Tanshinone IIA)	BMSC	Detection of GFP-expressing MSC	Oral administration to the rat model of acute myocardial infarction	Han et al., 2019

**TABLE 2:** Plant-derived components stimulate signaling for MSC migration.

**Component**	**MSC source**	**Effects on MSC migration**	**References**
Protocatechuic acid (PCA)	AMSC	Expression of MT1-MMP, and activation of MMP-2	Wang et al., 2008
Cinnamtannin B-1	BMSC	Activation of PI3K, PLC, lipoxygenase, and purines	Fujita et al., 2015
Cannabinoids	AMSC	Activation of Erk1/2 MAPK	Schmuhl et al., 2014
Icariin (ICA)	BMSC	Activation of MAPKs (p38, Erk1/2, JNK), and Expression of CXCR4	Jiao et al., 2018; Zhu et al., 2018
Tanshinone IIA (Tan IIA)	BMSC	Expression of CXCL12 (SDF-1) in target tissue, and CXCR4 in MSCs	Tong et al., 2011
Astragaloside IV (AS IV)	BMSC	Expression of CXCR4	Xie et al., 2013
Tetramethylpyrazine (TMP)	BMSC	Expression of CXCL12 (SDF-1) in target tissue, and CXCR4 in MSCs	Li et al., 2017a, 2019
Guanxin danshen formulation (GXDS) (Including Tanshinone IIA)	BMSC	Expression of CXCL12 (SDF-1) in target tissue	Han et al., 2019

### Protocatechuic Acid

The phenolic compound protocatechuic acid (PCA) has been isolated from a number of herbs and edible plants (Khan et al., 2015). PCA has been reported to have various pharmacological effects such as anti-oxidant, anti-inflammatory, anti-hyperglycemic, anti-apoptotic and antimicrobial activity. PCA has also been shown to inhibit the proliferation and migration of airway smooth muscle cells in tracheal inflammatory conditions (Liu Y. D. et al., 2019), while also suppressing the proliferation and migration of cardiac fibroblasts associated with p38 MAPK activation in a cardiac fibrosis model (Song and Ren, 2019).

Additionally, studies on MSC migration, using Transwell assays and gelatin-coated membranes, have shown that PCA isolated from the kernel of Alpinia oxyphylla (0.5 mM or more) enhanced adipose tissue-derived mesenchymal stem cell (AMSC) migration (Wang et al., 2008), which subsequently inhibited initiation of early apoptotic events. Furthermore, following PCA treatment, cell morphology and surface markers remained unchanged, and AMSCs retained their osteocyte, adipocyte, and cardiomyocyte-like cell differentiation potential properties. Although PCA has been shown to stimulate AMSC proliferation, while retaining their functional pluripotency (Wang et al., 2009), it has also been reported that PCA has a stimulatory effect on osteocyte differentiation and a suppressive effect on adipocyte differentiation in mouse embryo-derived MSC (Rivera-Piza et al., 2017).

The mechanism associated with PCA-stimulated MSC migration involves an increase in the expression of membrane-type matrix metalloproteinase-1 (MT1-MMP) and activation of matrix metalloproteinase-2 (MMP-2) in AMSCs. Since it has been reported that PCA activates three MAPKs (extracellular signal-regulated kinase1/2; Erk1/2, c-Jun N-terminal kinase; JNK and p38), which in turn activate the MMP signal in Schwann cell migration (Ju et al., 2015), PCA may serve to activate MAPKs involved in upstream signaling of MT1-MMP and MMP2 during AMSC migration. However, further studies are required to verify whether various doses of PCA activate MAPKs and promote migration of MSCs. MMPs play a key role in AMSC entry and migration through extracellular matrix (ECM) barriers, such as the basement membrane (Nitzsche et al., 2017). In the process of cell migration across the ECM barrier, PCA-stimulated AMSCs may promote pericellular ECM degradation by MMPs, thereby partially promoting cell migration. These studies provide evidence for the mechanism by which PCA may facilitate MSC migration to organs in MSC therapy.

### Cinnamtannin B-1

Cinnamtannin B-1 is a proanthocyanidin found in specific plants such as *Cinnamomum zeylanicum* (Sánchez-Rubio et al., 2018). Cinnamtannin B-1 exhibits anti-inflammatory, anti-oxidant and anti-thrombotic properties (López et al., 2008), and also protects neurons from ischemia/reperfusion-induced dysfunction (Chi et al., 2013), while inhibiting survival of cancer cells (Carriere et al., 2018), and protecting pancreatic acinar cells during pancreatitis (Rivera-Barreno et al., 2010).

*Mallotus philippinensis* bark extract also contains cinnamtannin B-1 (Furumoto et al., 2014). The use of this extract has been shown to increase migration of bone marrow-derived mesenchymal stem cells (BMSCs) and enhance wound healing in a mouse model (Furumoto et al., 2014). In fact, the efficacy of the extract on BMSC migration has been shown to be higher in comparison to other skin cell types. Histopathological features observed upon treatment with the extract indicated tissue repair by enhancement of angiogenesis and granulation tissue. *Mallotus philippinensis* fruit glandular hair also promoted wound healing via anti-oxidant and anti-inflammatory activity in a rat model (Gangwar et al., 2015).

Cinnamtannin B-1 has also been shown to promote BMSC migration when administered within the range of 0.8–2 μg/ml, and enhanced wound healing in a mouse model following topical administration (1.2 and 2.4 μg/wound) (Fujita et al., 2015). Further, examination of BMSC recruitment in blood circulation via cell marker analysis showed that mobilization of endogenous MSCs into the blood increased following cinnamtannin B-1 treatment. Image analysis of an animal model implanted with luciferase-expressing BMSC indicated that cinnamtannin B-1 increased BMSC accumulation at the wound site and promoted wound healing. Furthermore, cinnamtannin B-1-induced BMSC migration was attenuated by inhibitors of PI3K, phospholipase C (PLC), lipoxygenase (LOX), and purine, implicating the associated signaling pathways in BMSC migration. In addition, morelloflavone, a biflavonoid constituent from *Garcinia vilersiana* Pierre with structural similarities to cinnamtannin B-1, also enhanced BMSC migration with a similar pharmacological profile (Karanjgaokar et al., 1967). Therefore, structurally related compounds may also be effective in treating skin wounds by inducing mobilization of BMSCs.

### Cannabinoid

Cannabinoids are chemical components found in cannabis plants, with more than 100 varieties (Grof, 2018). The two primary types include cannabidiol (CBD) and tetrahydrocannabinol (THC), with the latter serving as the primary psychoactive component. CB1, CB2, TRPV1 and orphan G protein-coupled receptor GPR55, have been reported to exist as endogenous cannabinoid receptors (Hryhorowicz et al., 2018). Cannabinoids have a variety of effects, in addition to their analgesic properties (Kaur et al., 2016). Specifically, cannabinoids inhibit the growth and migration of gliomas (Vaccani et al., 2005), inhibit inflammation via neutrophil recruitment (Schicho et al., 2011), promote wound healing through cell adhesion as well as migration of fibroblasts and keratinocytes (Wang et al., 2016; Liu C. et al., 2019), and promote bone formation by osteoblast regulation (Idris and Ralston, 2012).

It has also been reported that CBD, administered within the range of 0.01–3 μM, increased AMSC migration in a dose-dependent manner (Schmuhl et al., 2014). However, CBD-induced AMSC migration was antagonized by inhibition of the Erk1/2 MAPK pathway via attenuated Erk1/2 phosphorylation in the presence of a CB2 receptor antagonist and GRP55 agonist. Conversely, Erk1/2-dependent migratory effects were observed following stimulating with CB2 receptor agonist and GPR55 antagonist. Thus, the basis for CBD-induced MSC migration involves activation of CB2 or inhibition of GPR55, which subsequently activates Erk1/2. Furthermore, long-term treatment with CBD has been shown to differentiate AMSCs into osteoblasts (Schmuhl et al., 2014). Hence, prolonged incubation of AMSC with CBD may enhance bone regeneration. In fact, recently, using a bone deficient rat model, it was reported that microspheres supplemented with CBD promoted the recruitment of MSCs and regeneration of bone defects (Kamali et al., 2019).

Similarly, the major psychoactive component, Δ9-tetrahydrocannabinol (Δ9-THC), also increased AMSC migration (Lüder et al., 2017). In the case of THC, migration of AMSCs is facilitated by CB1 through Erk1/2 activation, although THC has been reported to respond to both CB1 and CB2 (Pertwee, 2008). However, since cannabinoids can respond to many functional proteins, further research is needed to obtain a complete understanding of their pharmacological effects (Hryhorowicz et al., 2018).

### Icariin

Icariin (ICA) is a flavonoid glycoside isolated from *Epimedium* and is a primary component of the Chinese herb Herba Epimedii (Li et al., 2015). It has been reported that ICA possesses broad therapeutic activities, such as anti-oxidant, tonic, aphrodisiac, neuro-protective, cardio-protective, anti-rheumatic, anti-cancer, and anti-osteoporotic effects (Li et al., 2015). ICA has also been reported to promote BMSC proliferation, bone formation, and chondrogenic differentiation (Wang et al., 2014; Qin et al., 2015). Furthermore, treatment of AMSCs with ICA in a rat model of diabetes-related erectile dysfunction, protected against oxidative stress via PI3K signaling and improved AMSC survival, leading to enhanced therapeutic potential (Wang X. et al., 2017). Furthermore, combining ICA and MSCs was shown to promote angiogenesis and neurogenesis, as a result of increased production of VEGF and brain-derived neurotrophic factor (BDNF) through activation of PI3K and Erk1/2 MAPK, in an ischemic stroke rat model (Liu D. et al., 2018). ICA may also improve wound and periodontal pathology by promoting keratinocyte proliferation and migration, as well as by promoting survival and migration of periodontal ligament fibroblasts (Liu H. J. et al., 2018; Mi et al., 2018).

ICA administered at 1 μM was reported to significantly increase BMSC migration by stimulating actin stress fiber formation (Jiao et al., 2018). MAPK signals, such as p38, Erk1/2 and JNK, were activated during BMSC migration following ICA stimulation. In the presence of MAPK inhibitors, BMSC migration was also inhibited, and actin stress fiber formation was abolished. Thus, ICA may promote BMSC migration by increasing actin stress fiber formation through MAPK signaling. ICA also promotes CXCR4 expression, which is a signaling molecule upstream of MAPK, in BMSC migration through the activation of hypoxia inducible factor-1 (HIF-1) (Zhu et al., 2018). Moreover, transplantation of ICA-treated BMSCs in a cartilage-deficient rabbit model, accelerated the migration of BMSCs to the cartilage-deficient region in comparison to non-treated BMSCs (Jiao et al., 2018). These results suggest that ICA-treated BMSC may be effective in treating cartilage defects.

### Tanshinone IIA

Tanshinone IIA (Tan IIA) is a diterpene quinone and one of the major active compounds of *Salvia miltiorrhiza* (Lamiaceae) (Ren et al., 2019). *Salvia miltiorrhiza* has been widely used as a herbal medicine in the clinical treatment of cardiovascular diseases (Li et al., 2018a). Tan IIA has anti-oxidant and anti-inflammatory properties as well as multiple pharmacological benefits such as cardio-protection, neuroprotection, vascular protection, and anti-cancer effects (Li et al., 2018a; Ren et al., 2019). As for cell migration, Tan IIA has been shown to inhibit vascular endothelial cell proliferation and migration by inhibiting VEGF expression and its signaling pathways (Xing et al., 2015; Fan et al., 2017; Lee et al., 2017).

Tan IIA has been reported to have an effect on MSC differentiation including promoting osteogenic differentiation in BMSCs (Qian et al., 2015), cord blood MSCs (Heo et al., 2017), Wharton’s jelly MSCs (Cabrera-Pérez et al., 2019), and periodontal ligament stem cells (Liu X. et al., 2019). In addition, Tan IIA effectively induced the differentiation of cord blood MSCs into liver cells in a rat model of cirrhosis (Yang et al., 2015), BMSCs into neuronal cells in a rat spinal cord injury model (Zhang et al., 2018), and placental MSCs into cardiomyocytes *in vitro* (Li K. et al., 2018). Furthermore, it has been reported that Tan IIA combined with MSC treatment exhibited a neuronal protective effect via suppression of neuronal apoptosis in a vascular dementia rat model (Kong et al., 2017), as well as by suppressing amyloid-related protein production and inflammation in an Aβ25-35-induced AD rat model (Huang et al., 2019).

Additionally, administration of 1–4 μM Tan IIA has been shown to significantly enhance BMSC migration (Tong et al., 2011). In a rat model of acute myocardial infarction, oral administration of Tan IIA at 30 mg/kg/d combined with BMSC transplantation increased BMSC recruitment to the damaged area following myocardial infarction, resulting in restored normal function of the left ventricle (LV) systolic and end-diastolic pressure, as well as enhanced VEGF expression in the infarcted region. Immunological detection of the occlusion site also suggests that Tan IIA may promote CXCL12 expression in the infarcted area, while increasing CXCL12 levels in peripheral blood. However, Tan IIA-induced BMSC migration was inhibited by a CXCR4 blocker. Furthermore, Tan IIA treatment was shown to enhance CXCR4 expression in BMSCs. These results suggest that Tan IIA increases CXCL12 expression at the site of injury, and increases BMSC migration by enhancing CXCR4 expression in BMSCs. Thus, Tan IIA may be effective in treating ischemic heart disease by MSC therapy.

### Astragaloside IV

Astragaloside IV (AS IV) is an active saponin and the major active ingredient of Astragalus membranaceus, used in traditional Chinese medicine (Li et al., 2017b). Astragalus membranaceus, commonly used with *S. miltiorrhiza* including Tan IIA, is commonly used to enhance cardiovascular disease protection (Wang D. et al., 2017). Moreover, AS IV has demonstrated pharmacological action for cerebral injury, cardiovascular disease, liver, diabetic nephropathy, and cancer (Li et al., 2017b). It has also been shown to reduce infarct size and arrhythmias, while improving ventricular function in ischemic heart disease (Xu et al., 2007). In cell migration, AS IV treatment enhances the proliferation and migration of a human osteoblast-like cell line, which may be facilitated by activation of the Hedgehog signaling pathway (Guo et al., 2019). AS IV also inhibits proliferation and migration of human dermal vascular smooth muscle cells, stimulated by PDGF-BB secreted during vascular injury, through inhibition of p38 MAPK signaling (Chen et al., 2014). It has also been reported that AS IV and Tan IIA promote tubular structure formation of BMSC-derived endothelial cell cells, similar to that of blood vessels, via the expression of connexins and cell connection (Li et al., 2018b).

Lastly, BMSCs stimulated with AS IV, administered at 0.4 μg/ml, was reported to increase CXCR4 expression, indicating that BMSC migration to its ligand CXCL12 was enhanced (Xie et al., 2013). In addition, this enhanced migration was suppressed by CXCR4 inhibitors. Additionally, in a rat model of acute myocardial infarction, BMSCs stimulated with AS IV displayed increased homing to ischemic myocardial sites, suggesting that AS IV enhances BMSC recruitment via increased CXCR4 expression. Although the effect of AS IV on MSC migration was not as strong as that observed with Tan IIA, when the two components are administered together, a synergistic enhancement of BMSC migration was observed, hence this combinatorial strategy may increase the efficacy of MSC transplant therapy.

### Tetramethylpyrazine

Tetramethylpyrazine (TMP) is pyrazine and an alkaloid isolated from Rhizoma Chuanxiong (*Ligusticum wallichii*) (Zhao et al., 2016). TMP functions as a neuro-protective, anti-apoptotic, anti-cancer, vasodilator, and anti-inflammatory agent (Zhao et al., 2016). With respect to cell migration, TMP increases brain microvascular endothelial cell proliferation and migration by partially increasing VEGF secretion (Zhang et al., 2014), and promoting migration of the neural progenitor by inducing CXCL12 expression through activation of the PI3K pathway (Kong et al., 2016). Alternatively, TMP inhibits the migration of neutrophils activated by inflammation in a rat cerebral ischemia model, which was reported to involve Akt and Erk signaling (Chang et al., 2015).

TMP was also shown to enhance growth and neuronal differentiation of BMSCs. Further, it was suggested to have anti-aging effects on the nervous system (Song et al., 2019). A Chinese therapy, the Jiuxin pill, containing TMP and borneol, is known to promote exosome secretion from cardiac MSCs, which may have a positive therapeutic effect on heart disease (Ruan et al., 2018). In MSC migration, pretreatment with 10–200 μM TMP, causes increased BMSC migration in a dose-dependent manner (Li et al., 2017a). TMP also promoted CXCR4 expression, which was inhibited by a CXCR4 blocker. Intravenous administration of TMP-pretreated BMSCs into a cerebral ischemic rat model demonstrated improved neurological function and enhanced recruitment of BMSCs to cerebral ischemic sites. In addition, angiogenesis and the expression of both CXCL12 and CXCR4 were promoted at the ischemia site. In a separate study, the combination of transplanted BMSCs and intraperitoneal administration of TMP (40 mg/kg/d) in a cerebral ischemia model not only promoted the CXCL12/CXCR4 axis, but also regenerated blood vessels and nerves via enhancement of VEGF and BDNF expression, leading to functional recovery (Li et al., 2019). Taken together, these results show that the combination of MSCs and TMP may contribute to CXCL12/CXCR4 axis augmentation and neuronal regeneration, suggesting that they may be effective in treating cerebral ischemic injury.

### Guanxin Danshen Formulation

Guanxin Danshen (GXDS), a Chinese herbal medicine, is an effective formulation for the treatment of ischemic heart diseases (Deng et al., 2017). The GXDS formulation is comprised of three materials: *Salvia miltiorrhiza* (Lamiaceae), *Panax notoginseng* (Lamiaceae), and *Dalbergia odorifera* (Fabaceae). It contains Tanshinone IIA, salvianolic acid B (Wang et al., 2013), ginsenoside Rb1, ginsenoside RG1, notoginsenoside R1 (Wan et al., 2007), and flavanols (Liu et al., 2005).

GXDS has also proven effective in MSC treatments. For instance, oral administration of GXDS (100 mg/kg/d) in combination with BMSC transplantation in a rat model of acute myocardial infarction improved cardiac function of the LV ejection fraction, LV fractional shortening, and LV end-systolic volume (Han et al., 2019). In addition, GXDS administration in combination with BMSC transplantation not only reduced cell apoptosis detected by TUNEL staining, but also enhanced peri- and infarcted angiogenesis, increased local CXCL12 expression and the number of BMSCs homing to the infarcted area, while also reducing the size of the myocardial infarction region. Therefore, it is suggested that GXDS increases the migration of MSCs by up-regulating the expression of CXCL12 at the site of infarction. However, since this formulation may contain Tan IIA, it is possible that this component could have contributed to the observed effects (Tong et al., 2011). Nevertheless, the combination of GXDS and MSC therapy has the potential to improve ischemic heart disease, including myocardial infarction.

Moreover, other Chinese herbal extracts have been shown to stimulated MSC migration. For example, oily extract from *Catharmus tinctorius* (1–50 μg/ml) promotes rat BMSC migration through Rho-associated protein kinase 2 (ROCK2) signaling *in vitro* (Liu X. et al., 2019). In addition, Bushen Huoxue decoction (100 μg/ml), a mixture comprised of eleven Chinese herbs, increase rat BMSC migration *in vitro* by regulating MiR-539-5p miRNA (Hu et al., 2019) and activating Wnt5a-related cell motility (Shen et al., 2018). Despite these observations, none of the individual compounds responsible have been identified from these extracts.

## Discussion

This article focused on a comprehensive review of plant-derived components that increase MSC migration and promote recovery from tissue damage. Studies on the promotion of MSC migration by plant-derived components have exposed a variety of characteristics and advantages (Tables 1, 2). For example, although PCA was only found to be effective at high doses, its potential has been demonstrated and has been suggested to function by increasing the expression and activation of MMPs, which then degrade the ECM to enhance MSC invasion into tissues (Wang et al., 2008). Studies on cinnamtannin B-1 have shown that structurally related flavonoids also have MSC migration activity (Fujita et al., 2015). Thus, expanding upon this structural framework offers the potential to develop more active compounds through structure-based drug development. Since CBD, a non-psychoactive cannabinoid, can promote MSC migration and bone differentiation, and is currently being therapeutically developed, for example in sustained-release drugs from the encapsulated microspheres of biodegradable polymers, it represents a potentially advanced option for bone repair (Schmuhl et al., 2014; Kamali et al., 2019). ICA, when used to pretreat MSCs, promote their migration to cartilage-defect sites (Jiao et al., 2018; Zhu et al., 2018), as well as their proliferation (Qin et al., 2015), bone differentiation (Li et al., 2015), and cartilage differentiation (Wang et al., 2014), and thus, may significantly improve healing related to skeletal defects. Tan IIA has been shown to increase CXCL12 expression at the site of injury as well as CXCR4 on transplanted MSCs, leading to enhanced MSC migration to the injured area, which may prove effective in the treatment of ischemic heart disease (Tong et al., 2011). Interestingly, AS IV and Tan IIA were found to synergistically enhance MSC migration, thus presenting potential for combined therapeutic approaches involving plant-derived components (Xie et al., 2013). The use of TMP in combination with MSCs, or as a pretreatment agent, not only promotes the migration of MSCs to the site of injury, but also enhances the effect of MSCs on the regeneration of nerves and blood vessels (Li et al., 2017a, 2019). Consequently, TMP has potential for the treatment of cerebral ischemia damage. Finally, GXDS, when administered orally, in combination with MSC transplantation, may improve acute myocardial infarction by reducing apoptosis at the site of injury, generating new blood vessels, and promoting MSC migration by increasing CXCL12 expression (Han et al., 2019).

The diverse array of mechanisms by which plant-derived components facilitate MSC migration include ECM remodeling, activation of intracellular signaling pathways, such as PI3K and MAPK, and enhanced expression of the CXCL12/CXCR4 axis (Figure 1 and Table 2). The plants from which these compounds have been isolated have been used in traditional medicine practices and as such, a significant amount of useful information is available with regard to their use in several indications. Many associated factors have already been analyzed in detail, including their identification, structural classification and therapeutic efficacy. Since each plant-derived component exhibits therapeutic effects in specific conditions, the disease models are designed based on the indication of the components, expecting the synergistic effects in MSC therapy (Table 3). Consequently, these disease models have demonstrated the benefits of MSC treatment as well as the additional therapeutic effects of many plant-derived components. Furthermore, medicinal plants have many uses and often have the advantage of being administered orally. The methods of administering plant-derived components *in vivo* for MSC migration enhancement include MSC pretreatment, as well as transdermal, intravenous, intraperitoneal and oral approaches.

**TABLE 3:** Therapeutic effects of plant-derived components and their effects on MSCs.

**Component**	**Plant**	**Therapeutic effects of component**	**Effects of component on MSC**	**Therapeutic model by component and MSC transplantation**
Protocatechuic acid (PCA)	Alpinia oxyphylla (Zingiberaceae) Khan et al., 2015	Anti-oxidant, Anti-inflammatory, Anti-hyperglycemic, Anti-apoptotic, Antimicrobial Khan et al., 2015, Inhibition of the proliferation and migration of airway smooth muscle cells in tracheal inflammatory conditions Liu Y. D. et al., 2019, Suppression of the proliferation and migration of cardiac fibroblasts in a cardiac fibrosis Song and Ren, 2019, Activation of the MMP signal in Schwann cell migration Ju et al., 2015	Migration Wang et al., 2008, Proliferation Wang et al., 2009, Stimulatory on osteocyte differentiation and suppressive on adipocyte differentiation Rivera-Piza et al., 2017	–
Cinnamtannin B-1	Mallotus philippinensis (Euphorbiaceae) Furumoto et al., 2014	Anti-oxidant, Anti-inflammatory, Anti-thrombotic López et al., 2008, Neuro-protective Chi et al., 2013, Pancreatic-protective Rivera-Barreno et al., 2010, Anti-cancer Carriere et al., 2018, Wound healing Gangwar et al., 2015	Migration Furumoto et al., 2014; Fujita et al., 2015	Wound healing Fujita et al., 2015
Cannabinoids	Cannabis sativa (Cannabaceae) Grof, 2018	Analgesic effects Kaur et al., 2016, Anti-inflamatory Schicho et al., 2011, Anti-gliomas Vaccani et al., 2005, Wound healing Wang et al., 2016; Liu C. et al., 2019, Bone formation Idris and Ralston, 2012	Migration Schmuhl et al., 2014; Lüder et al., 2017, Osteocyte differentiation Schmuhl et al., 2014	Bone-defect Kamali et al., 2019
Icariin (ICA)	Epimedium (Berberidaceae) Li et al., 2015	Anti-oxidant, Tonic, Aphrodisiac, Neuro-protective, Cardio-protective, Anti-rheumatic, Anti-cancer, Anti-osteoporotic Li et al., 2015, Wound healing by effects for keratinocyte proliferation and migration Mi et al., 2018, Improvement of periodontal pathology by effects for survival and migration of periodontal ligament fibroblasts Liu H. J. et al., 2018	Migration Jiao et al., 2018; Zhu et al., 2018, Proliferation, Osteocyte differentiation Qin et al., 2015, Chondrogenic differentiation Wang et al., 2014, Cell survival Wang X. et al., 2017, Neuronal regeneration in cooperation with MSCs Liu D. et al., 2018	Cartilage-defect Jiao et al., 2018, Diabetes-associated erectile dysfunction Wang X. et al., 2017, Cerebral ischemia Liu D. et al., 2018
Tanshinone IIA (Tan IIA)	Salvia miltiorrhiza (Lamiaceae) Ren et al., 2019	Anti-oxidant, Anti-inflammatory, Cardio-protective, Neuro-protective, Vascular- protective, Anti-cancer Li et al., 2018a; Ren et al., 2019 Inhibition of vascular endothelial cell proliferation and migration in angiogenesis-related pathologies Xing et al., 2015; Fan et al., 2017; Lee et al., 2017	Migration Tong et al., 2011, Osteogenic differentiation Qian et al., 2015; Heo et al., 2017; Cabrera-Pérez et al., 2019; Liu X. et al., 2019, Hepatocyte differentiation Yang et al., 2015, Neuronal differentiation Zhang et al., 2018, Cardiomyocyte differentiation Li K. et al., 2018, Neuro-protection in cooperation with MSCs Kong et al., 2017; Huang et al., 2019	Acute myocardial infarction Tong et al., 2011 Liver cirrhosis Yang et al., 2015, Spinal cord injury Zhang et al., 2018, Vascular dementia Kong et al., 2017, Aβ25-35-induced AD Huang et al., 2019
Astragaloside IV (AS IV)	Astragalus membranaceus (Leguminosae) Li et al., 2017b	Anti-oxidant, Anti-inflammatory, Cardio-protective, Neuro-protective, Hepato-protective, Nephro-protective, Anti-cancer Li et al., 2017b, Improvement of ventricular function in ischemic heart disease Xu et al., 2007, Enhancement of the proliferation and migration of a human osteoblast-like cell Guo et al., 2019, Inhibition of the abnormal proliferation and migration of human dermal vascular smooth muscle cells Chen et al., 2014	Migration Xie et al., 2013, Angiogenesis of MSC-derived endothelial cell-like cells by co-stimulation with Tan IIA Li et al., 2018b	Acute myocardial infarction Xie et al., 2013
Tetramethylpyrazine (TMP)	Rhizoma Chuanxiong (Ligusticum wallichii) Zhao et al., 2016	Anti-oxidant, Anti-inflammatory, Neuro-protective, Anti-apoptotic, Anti-cancer, Vasodilator Zhao et al., 2016, Increase of brain microvascular endothelial cell proliferation and migration Zhang et al., 2014, Promotion of migration of the neural progenitor Kong et al., 2016, Inhibition of the migration of neutrophils activated by inflammation Chang et al., 2015.	Migration Li et al., 2017a, 2019, Neuronal differentiation Song et al., 2019, Jiuxin pill containing TMP promotes exosome secretion from MSCs Ruan et al., 2018 Angiogenesis in cooperation with MSCs Li et al., 2017a, 2019 Neurogenesis in cooperation with MSCs Li et al., 2019	Cerebral ischemia Li et al., 2017a, 2019
Guanxin danshen formulation (GXDS) (Including Tanshinone IIA)	Salvia miltiorrhiza (Lamiaceae), Panax notoginseng (Lamiaceae), Dalbergia odorifera (Fabaceae) Deng et al., 2017	Cardio-protective Deng et al., 2017	Migration Han et al., 2019	Acute myocardial infarction Han et al., 2019

Mesenchymal stem cells are believed to play an important role in tissue regeneration after organ damage, and MSC-based therapeutic approaches have been implemented in a variety of disease models (Pittenger et al., 2019). Methods to control the kinetics of transplanted MSCs can be very useful in a variety of applications, including organ regeneration, protection from tissue damage, and treatment of refractory cancers (Park et al., 2015; Becker and Riet, 2016). Herein, we have provided a comprehensive review of plant-derived components that control the migration of MSCs, and may offer novel therapeutic options for regenerative medicine.

### Challenges and Future Prospects

Currently, a gap exists between cell-level migration and *in vivo* recruitment. Additionally, several issues have been described including, how to achieve specificity and effective dosing for MSC *in vivo*, how to confirm component toxicity, and how to address the limitations of MSC dynamic tracking technology *in vivo* for cell kinetic analysis of MSC transplant (Krueger et al., 2018). To solve these issues, tests for drug development must determine the tissue specificity and effective dose of each component *in vivo*, as well as analyze individual toxicity.

As a supplement to cell dynamic analysis, it is also necessary to accumulate omics information on the effects of plant-derived components on MSC mobilization in disease models. For example, with the progress of MSC research, analysis of single cells using techniques such as RNA-Seq, will allow for examination of detailed cellular functions along with the MSC fates at the genetic level (Liu S. et al., 2019). Moreover, the effects and suitability of plant-derived components in various MSCs at the genetic level may be elucidated. Furthermore, it has been shown that exosomes secreted by MSCs have a healing effect, and contain a large number of biomolecules such as miRNAs, cytokines, growth factors and enzymes, thereby demonstrating that advances are being made in defining the molecular basis for the therapeutic action of MSCs (Yin et al., 2019).

## Conclusion

Plant-derived components, which promote MSC migration, accelerate the healing of tissue damage. Moreover, MSC migration by plant-derived components may be mediated by signaling molecules such as CXCL12/CXCR4, PI3K, MAPK, and MMPs. Ingredients derived from medicinal plants are useful as there already exists invaluable information on medicinal plants for traditional use. In addition, the structural characteristics of plant-derived components are important to understand their effect on MSC migration activity, and may be potential seeds for drug discovery. Therefore, plant-derived components that enhance MSC recruitment to damaged sites may provide novel tools for improved treatment approaches.

## Author Contributions

AM contributed to the design and implementation of the research and writing of the manuscript.

## Conflict of Interest

The author declares that the research was conducted in the absence of any commercial or financial relationships that could be construed as a potential conflict of interest.
